# Novel myocardial markers *GADD45G and NDUFS5 i*dentified by RNA-sequencing predicts left ventricular reverse remodeling in advanced non-ischemic heart failure: a retrospective cohort study

**DOI:** 10.1186/s12872-020-01396-2

**Published:** 2020-03-05

**Authors:** Togo Iwahana, Sho Okada, Masato Kanda, Motohiko Oshima, Atsushi Iwama, Goro Matsumiya, Yoshio Kobayashi

**Affiliations:** 1 Department of Cardiovascular Medicine, Chiba University Graduate School of Medicine, 1-8-1 Inohana, Chuo-ku, Chiba, 260-8670 Japan; 2 Department of Cellular and Molecular Medicine, Chiba University Graduate School of Medicine, Chiba, Japan; 3 Department of Cardiovascular Surgery, Chiba University Graduate School of Medicine, Chiba, Japan

**Keywords:** NIDCM, LVRR, RNA-seq, NDUFS5, GADD45G

## Abstract

**Background:**

Left ventricular reverse remodeling (LVRR) has been detected in non-ischemic dilated cardiomyopathy (NIDCM) patients following optimal treatment. However, its prediction with only conventional modalities is often difficult. This study sought to examine whether RNA sequencing (RNA-seq) of myocardium tissue samples could predict LVRR in NIDCM.

**Methods:**

A total of 17 advanced NIDCM patients with left ventricular ejection fraction (LVEF) below 30% who underwent cardiac biopsy from Left ventricle (LV) were prospectively recruited. They received optimal treatment and followed with echocardiogram every 6 months. Based on LVRR status after 12 months of treatment, patients were divided into the reverse remodeling (RR) or non-RR group. Tissue samples were analyzed by RNA-seq, and a functional analysis of differentially expressed genes was carried out.

**Results:**

There were eight and nine patients in the RR and non-RR groups, respectively. No difference was found in age, sex, disease duration, LV end-diastolic diameter, and LVEF between the two groups. There were 155 genes that were differentially expressed between the two groups. *Nicotinamide adenine dinucleotide ubiquinone oxidoreductase subunit* (*NDUF*)*S5* and *Growth arrest and DNA-damage-inducible protein* (*GADD*)*45G*, along with several genes related to the mitochondrial respiratory chain and ribosome, were significantly downregulated in the RR as compared to the non-RR group.

**Conclusion:**

*GADD45G* and *NDUFS5* are potential biomarkers for LVRR in patients with advanced NIDCM.

## Background

Heart failure has a complex etiology and is associated with various conditions, and is a major cause of adult mortality. In particular, non-ischemic dilated cardiomyopathy (NIDCM)—which is characterized by left ventricular (LV) dilation and a severely reduced LV ejection fraction (LVEF) without coronary artery disease—is often refractory to established drugs such as β-blockers, angiotensin-converting enzyme inhibitors, and angiotensin II receptor blockers and to cardiac resynchronization therapy (CRT). Therefore, NIDCM often requires heart transplantation or ventricular assist device (VAD) implantation. However, the former is available only to a limited number of patients, whereas the latter is associated with complications like stroke, bleeding, and infection that can be fatal during the long waiting period for transplantation [1].

On the other hand, many NIDCM patients show recovery of cardiac function after optimal pharmacological or device therapy [2, 3]. This phenomenon, known as LV reverse remodeling (LVRR), is associated with improved clinical outcomes. Accordingly, several indices have been identified as predictors of LVRR and heart failure, including blood biomarkers like suppression of tumorigenesis-2 and galectin-3 [4–8], late gadolinium enhancement by cardiac magnetic resonance imaging [9], cardiac fibrosis and cardiomyocyte hypertrophy by histopathological examination [10, 11], and washout rate in meta-iodobenzylguanidine myocardial scintigraphy [12]. However, conventional modalities cannot accurately predict the occurrence of LVRR.

Analyzing myocardial gene expression can be more useful for this purpose. Several studies have demonstrated a clear correlation between myocardial expression of certain mRNAs or micro (mi)RNAs and LVRR in NIDCM patients by quantitative reverse transcription PCR [13, 14]. Moreover, RNA sequencing (RNA-seq) and next generation sequencing (NGS)—which enables higher throughput and greater accuracy in the evaluation of gene expression than microarray analysis [15]—is increasingly used in clinical settings [16]. Previous NGS studies have revealed differences in gene expression profiles between ischemic and non-ischemic cardiomyopathy [17, 18] or pre- and post VAD support [19–21]. However, there have been no studies using comprehensive transcriptome analysis to examine specific genes related to LVRR. The present study therefore aimed to identify factors that can predict LVRR occurrence by RNA-seq.

## Methods

### Patient selection

Patients with advanced NIDCM whose LVEF was < 30% by echocardiogram and who underwent cardiac biopsy of the LV at Chiba University Hospital from September 2014 to May 2016 were included in the study. Exclusion criteria were as follows: (i) heart failure with ischemic etiology (defined as past history of myocardial infarction or significant stenosis in the main branches of coronary arteries); (ii) inflammatory or infiltrative heart disease including myocarditis, sarcoidosis, or amyloidosis; (iii) other secondary or metabolic cardiomyopathies such as neuromuscular disease and alcoholic cardiomyopathy; (iv) dependence on hemodialysis; and (v) age younger than 20 years or older than 65 years.

### Patient follow-up

Prior to myocardial biopsy, all patients underwent blood sample collection, echocardiogram, and right heart catheterization. After myocardial biopsy, patients continued to receive optimal treatment including guideline-directed medical therapy with β-blockers and renin-angiotensin blockers and CRT. Patients were followed for 6 to 12 months, with echocardiogram performed at each follow up; they were then divided into the reverse remodeling (RR) and non-RR groups according to positive or negative LVRR status, respectively. LVRR was defined as (i) LVEF improvement of > 10% in absolute value, and (ii) LVEF > 30%.

### RNA extraction and cDNA library preparation

LV myocardium samples were collected at the time of cardiac catheter examination or open heart surgery, including for VAD implantation and valve replacement. Samples were immediately treated with RNAlater RNA stabilization reagent (Qiagen, Valencia, CA, USA) and stored at − 20 °C. Total RNA was extracted using mirVana miRNA Isolation kit (Life Technologies, Carlsbad, CA, USA) according to the manufacturer’s instructions, and cDNA libraries were generated using a NEBNext Ultra RNA Library Prep kit (New England BioLabs, Beverly, MA, USA). Sequencing was performed using a HiSeq1500 system (Illumina, San Diego, CA, USA) with a single-read sequencing length of 60 bp.

### RNA-seq data analysis

TopHat v.1.3.2 with default parameters was used to map sequences against the UCSC/hg19 reference genome. Gene expression level was quantified using Cufflinks v.2.0.2 [22, 23] as fragments per kilobase of exon per million fragments (FPKM) mapped reads. In the present study, miRNAs and genes expressed at low levels (FPKM < 1 in all samples) were eliminated. To identify novel biomarkers that predict the occurrence of LVRR, we carried out sensitivity, specificity, and receiver operating characteristic (ROC) curve analyses for each differentially expressed gene (DEG). Functional annotation was performed using the Database for Annotation, Visualization, and Integrated Discovery [24–26]. Gene expression profiles of two groups were compared by principal component analysis (PCA). RNA-sequencing data was deposited in the DNA Data Bank of Japan (accession number PRJDB5368).

### Quantitative real-time PCR

When the same cDNAs as for RNA-seq were available, quantitative real-time PCR (qPCR) was performed on a Light Cycler 480 instrument (Roche, Basel, Switzerland) using the Taqman Universal Probe Library and the Light Cycler 480 Probes Master (Roche, Basel, Switzerland) according to the manufacturer’s instruction. 18S ribosomal RNA was used to normalize the RNA content of the samples. Obtained results were expressed as relative mRNA levels of the cycle threshold value, which was then converted to fold change. Designed primer pairs and probe number are as follows: *NDUFS5*, 5′-gatttcgtagagtgtttgcttcg-3′ (F), 5′-gaggtggaggggtgtactttc-3′ (R), probe number #38 (catalog number 04687965001); GADD45G, 5′-cagccaaagtcttgaacgtg-3′ (F), 5′-cctggatcagcgtaaaatgg-3′ (R), probe number #71 (catalog number 04688945001).

### Statistical analysis

For patients’ clinical data, continuous variables are expressed as the mean ± standard deviation and were compared with the unpaired t test, whereas categorical data are expressed as a percentage and were compared with the χ^2^ test. *P* < 0.05 was considered statistically significant. DEGs were defined as those with a significance (*P*) value < 0.05, false discovery rate < 5%, and fold change > 1.5, which was determined using the R v.3.1.0 software tag count comparison function [27]. The significance value for functional annotation analysis was *P* < 0.05. Data were analyzed using R version 3.3.2 (The R Foundation, Vienna, Austria).

Logistic regression analysis was used to assess the impact of selected DEGs on LVRR. Odds ratios (OR) and 95% confidence intervals (CI) were calculated. Confounders with *P* values < 0.05 in the univariate analysis were entered into the multivariate model. A *P* value < 0.05 was regarded as statistically significant. Data were analyzed using STATA version 15.1 (StataCorp, College Station, TX, USA).

## Results

### Patient characteristics

A total of 20 patients were enrolled in this study. Two were excluded due to insufficient RNA amount and heart transplantation in the prior 6 months, respectively. One patient diagnosed with acute fulminant myocarditis was also excluded. Ultimately, 17 patients (3 females and 14 males, mean age: 46.6 ± 11.6 years) without coronary artery disease or other secondary or specific cardiomyopathies were analyzed. LV myocardial samples were obtained by transcatheter biopsy for four patients, by needle biopsy during aortic valve replacement surgery for three patients, or during VAD implantation for 10 patients.

Patients were divided into RR (*n* = 8) and non-RR (*n* = 9) groups based on the findings of a follow-up echocardiogram. There were no differences between the two groups in terms of age, sex, disease duration, LVEF on the echocardiogram, brain natriuretic peptide level, cardiac index for right heart catheterization, or ratio of cardioprotective medications (Table 1). On the other hand, proportion of surgical biopsy, inotropes used, and VAD implantation, and serum creatinine concentration were significantly higher in the non-RR than in the RR group (Table 1). Disease duration was more than double in the non-RR group but did not reach statistical significance.

**Table 1:** Baseline characteristics of the patients

variables	RR group (*n* = 8)	non-RR group (*n* = 9)	*P* value
Age	46.1 ± 14.3	47.1 ± 9.4	0.87
Male Sex, n	6	8	0.45
Disease duration (months)	36.1 ± 65.5	79.0 ± 46.5	0.14
Height (cm)	165 ± 9	168 ± 5	0.35
Weight (kg)	64.1 ± 12.5	67.2 ± 9.4	0.56
BMI	23.4 ± 3.9	23.8 ± 3.2	0.83
HR (bpm)	90 ± 23	84 ± 20	0.63
Method of biopsy (transcatheter /surgery)	4/4	0/9	0.019
Treatment			
Beta-blocker, n	7	8	0.93
ACEI or ARB, n	6	8	0.45
Inotropes, n	2	8	0.008
IABP, n	0	2	0.16
AVR, n	2	1	0.45
VAD implantation, n	2	8	0.008
Labo data			
Hb (g/dL)	12.8 ± 2.3	11.4 ± 1.7	0.17
T-Bil (mg/dl)	1.1 ± 0.3	1.2 ± 0.8	0.70
Cre (mg/dl)	0.92 ± 0.36	1.40 ± 0.45	0.029
BNP (pg/ml)	725 ± 454	475 ± 177	0.15
Echocardiogram			
LVDD (mm)	72 ± 10	79 ± 12	0.19
LVDS (mm)	65 ± 8	72 ± 12	0.20
LVEF (%)	21.5 ± 5.1	20.4 ± 4.2	0.63
LAD (mm)	50 ± 9	54 ± 11	0.43
AR (moderate to severe, n)	2	1	0.45
MR (moderate to severe, n)	6	6	0.71
TR (moderate to severe, n)	2	3	0.71
Hemodynamics			
PAP, mean (mmHg)	36 ± 10	38 ± 10	0.60
PCWP, mean (mmHg)	27 ± 6	29 ± 9	0.57
RAP, mean (mmHg)	10 ± 2	13 ± 6	0.16
CO (L/min)	4.02 ± 0.68	3.79 ± 0.90	0.56
CI (L/min/m^2^)	2.30 ± 0.34	2.06 ± 0.47	0.25
PVR (Wood)	2.41 ± 1.66	2.45 ± 1.61	0.96

Data are shown as the mean value ± standard deviation, or number of the patients

Abbreviations: *BMI* body mass index; *HR* heart rate; *ACEI* angiotensin converting enzyme inhibitor; *ARB* angiotensin II receptor blocker; *IABP* intraaortic balloon pumping; *AVR* aortic valve replacement; *VAD* ventricular assist device; *Hb* hemoglobin; *T-Bil* total bilirubin; *Cre* creatinine; *LVDD* left ventricular diastolic diameter; *LVDS* left ventricular systolic diameter; *LVEF* left ventricular ejection fraction; *LAD* left atrial diameter; *AR* aortic regurgitation; *MR* mitral regurgitation; *TR* tricuspid regurgitation; *PAP* pulmonary artery pressure; *PCWP* pulmonary capillary wedge pressure; *RAP* right atrial pressure; *CO* cardiac output; *CI* cardiac index; *PVR* pulmonary vascular resistance

### RNA-seq analysis

**Fig. 1:**
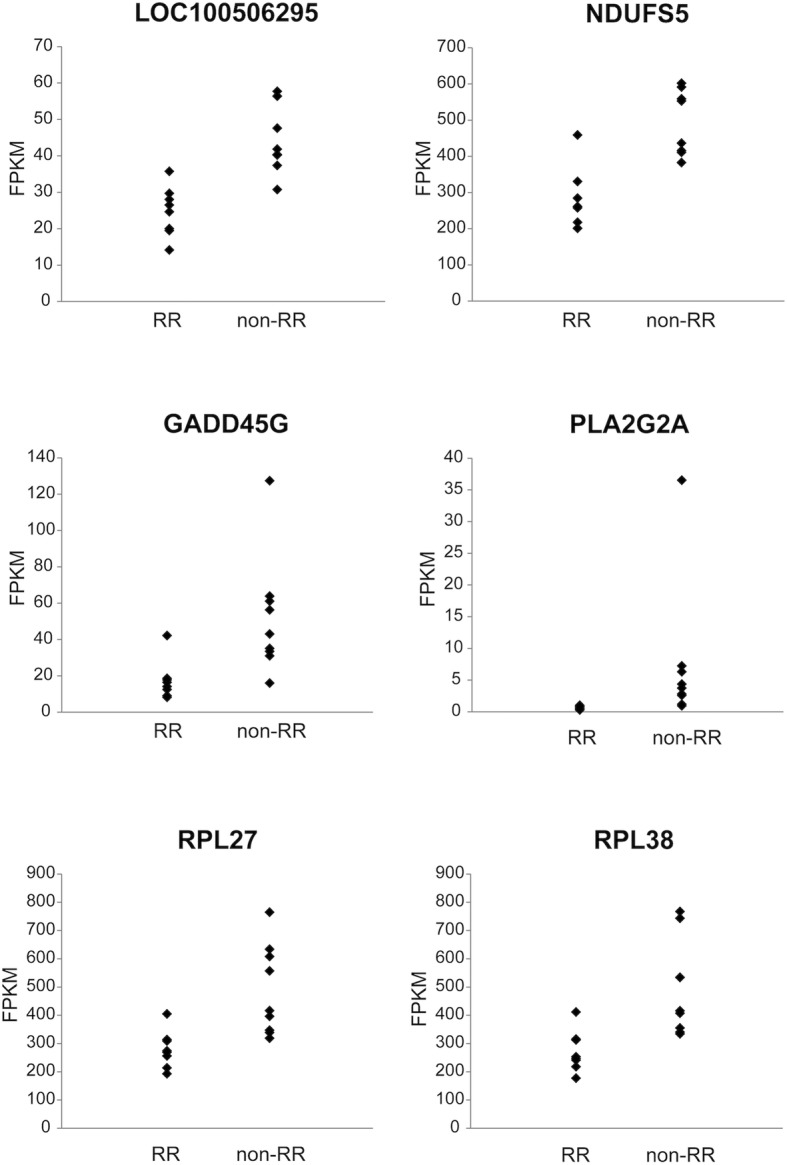
Scatterplots of the top six genes differentially expressed between RR and non-RR groups. The range of expression is shown as a scatterplot for the top six DEGs (*n* = 8 and 9 in the RR and non-RR groups, respectively). DEG, differentially expressed gene; RR, reverse remodeling; FPKM, Fragments per kilobase of exon per million fragments mapped reads

**Fig. 2:**
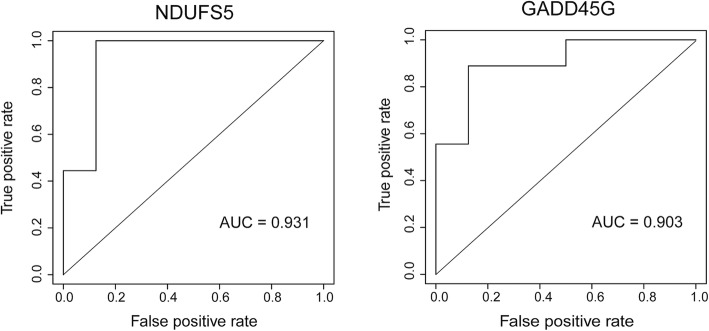
ROC curve analysis to predict LVRR. Both *NDUFS5* and *GADD45G* showed high predictive capacity for LVRR (*n* = 8 and 9 in the RR and non-RR groups, respectively). ROC, receiver operating characteristic; AUC, area under the curve; LVRR, LV reverse remodeling

**Fig. 3:**
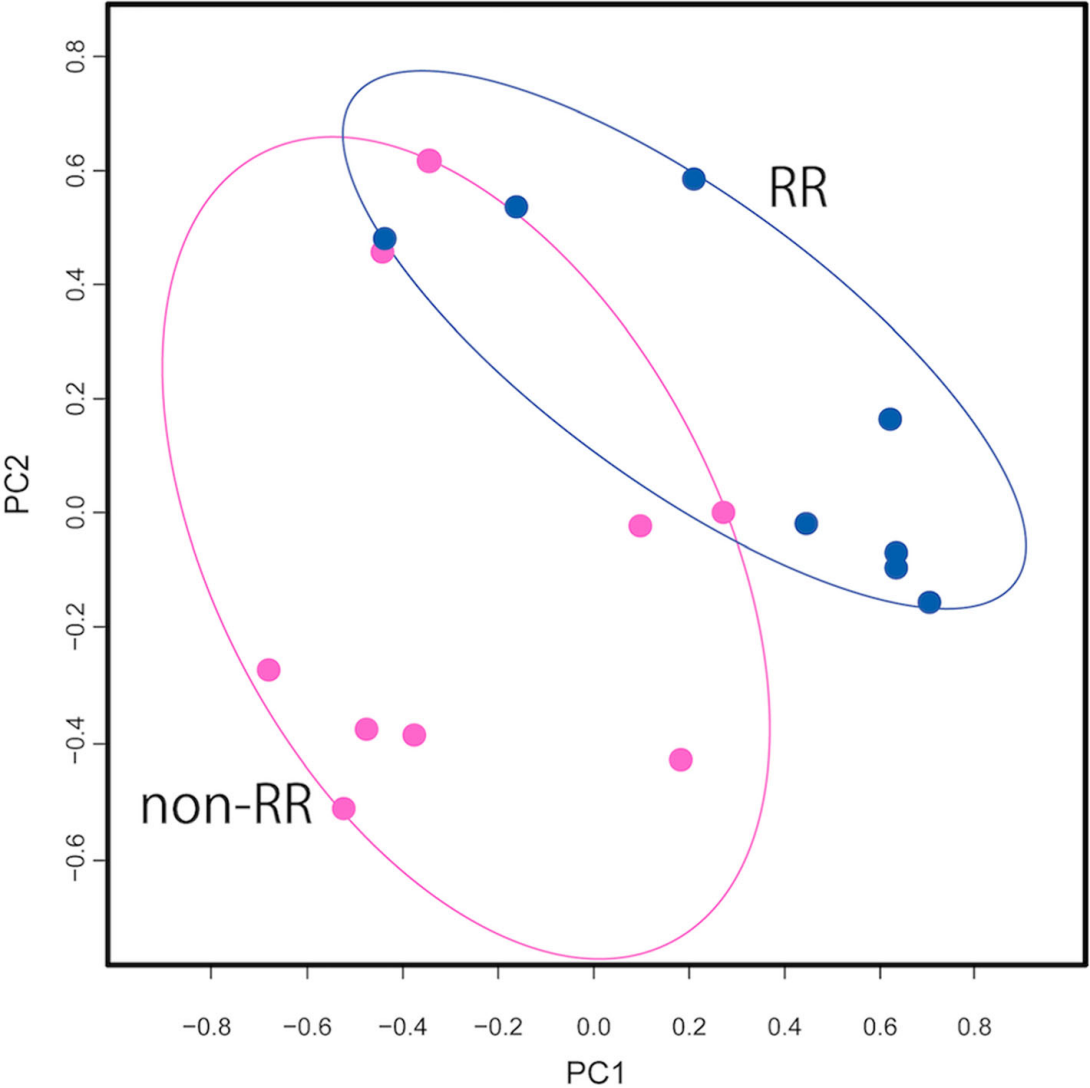
PCA of distributions in RR and non-RR groups. Distributions differed between RR and non-RR groups (*n* = 8 and 9 in the RR and non-RR groups, respectively). RR, reverse remodeling

A total of 22,416 genes were detected by RNA-seq. After excluding miRNAs and genes with low expression (FPKM < 1 in each sample), 14,448 genes were retained for analysis. Of these, 155 were differentially expressed between the RR and non-RR groups, with 150 genes upregulated in the non-RR group (Table 2). The top three DEGs were *LOC100506295*, *Nicotinamide adenine dinucleotide ubiquinone oxidoreductase subunit* (*NDUFS*)*5*, and *Growth arrest and DNA-damage-inducible protein* (*GADD*)*45G* (Fig. 1). Genes encoding other NDUF subunits (*NDUFB1*, *NDUFA1*, *NDUFS6*, *NDUFA13*, *NDUFA3*, *NDUFB7*, and *NDUFB3*) as well as various ribosomal proteins were also upregulated. ROC curve analysis revealed that the three genes (*LOC100506295*, *NDUFS5*, and *GADD45G*) had high predictive capacity for LVRR (Fig. 2; areas under the curve = 0.99, 0.93, and 0.90, respectively). A functional enrichment analysis showed significant enrichment of genes related to the mitochondrial respiratory chain or ribosome (Table 3). The PCA showed separate distributions for the RR and non-RR groups (Fig. 3).

**Table 2:** Top 20 differentially expressed genes

Rank	Gene symbol	Official full name	*P* value	FDR	Fold change
1	*LOC100506295*		6.69E−07	0.0097	1.8320
2	*NDUFS5*	NADH: ubiquinone oxidoreductase subunit S5	1.51E−06	0.0109	1.7411
3	*GADD45G*	Growth arrest and DNA damage-inducible gamma	2.26E−06	0.0109	2.9929
4	*PLA2G2A*	Phospholipase A2 group IIA	3.69E− 06	0.0125	10.8842
5	*RPL27*	Ribosomal protein L27	7.84E−06	0.0125	1.7440
6	*RPL38*	Ribosomal protein L38	9.35E−06	0.0125	1.8101
7	*RPS9*	Ribosomal protein S9	9.37E−06	0.0125	1.6479
8	*LOC100506167*		9.61E−06	0.0125	12.2276
9	*RPL35*	Ribosomal protein L35	1.36E−05	0.0125	1.7464
10	*C11orf10*	Transmembrane protein 258	1.50E− 05	0.0125	1.6109
11	*RPS19*	Ribosomal protein S19	1.54E− 05	0.0125	1.6684
12	*RPS24*	Ribosomal protein S24	1.69E−05	0.0125	1.6003
13	*RPL31*	Ribosomal protein L31	1.71E−05	0.0125	1.7753
14	*ATP5EP2*	ATP synthase, H+ transporting, mitochondrial F1 complex, epsilon subunit pseudogene 2	1.83E−05	0.0125	1.6903
15	*NDUFB1*	NADH: ubiquinone oxidoreductase subunit B1	1.93E−05	0.0125	1.8706
16	*CFD*	Complement factor D	2.10E−05	0.0125	2.7821
17	*RPS21*	Ribosomal protein S21	2.10E−05	0.0125	1.7684
18	*OST4*	Oligosaccharyltransferase complex Subunit 4, non-catalytic	2.14E−05	0.0125	1.6003
19	*POLR2L*	RNA polymerase II subunit L	2.37E−05	0.0125	1.5835
20	*C14orf2*	Chromosome 14 open reading frame 2	2.45E−05	0.0125	1.7078

*FDR* false discovery rate

**Table 3:** Functional enrichment analysis results

Term	*P*-Value	Fold Enrichment	FDR
GO:0006614 SRP-dependent cotranslational protein targeting to membrane	5.13E-91	50.93	7.89E-88
GO:0019083 viral transcription	1.22E-84	42.89	1.87E-81
GO:0000184 nuclear-transcribed mRNA catabolic process, nonsense-mediated decay	1.47E-82	40.41	2.26E-79
GO:0006413 translational initiation	6.55E-78	35.18	1.01E-74
GO:0003735 structural constituent of ribosome	8.01E-78	24.63	1.03E-74
GO:0006412 translation	2.00E-72	21.82	3.08E-69
GO:0005840 ribosome	4.28E-72	29.64	5.41E-69
GO:0006364 rRNA processing	2.87E-64	22.53	4.42E-61
GO:0022625 cytosolic large ribosomal subunit	1.80E-45	40.55	2.27E-42
GO:0022627 cytosolic small ribosomal subunit	2.08E-38	47.97	2.62E-35
GO:0005743 mitochondrial inner membrane	1.61E-33	9.47	2.04E-30
GO:0044822 poly(A) RNA binding	1.81E-24	4.46	2.34E-21
GO:0005747 mitochondrial respiratory chain complex I	9.49E-23	32.38	1.20E-19
GO:0015935 small ribosomal subunit	1.30E-22	49.48	1.64E-19
GO:0006120 mitochondrial electron transport, NADH to ubiquinone	1.39E-22	31.60	2.14E-19
GO:0008137 NADH dehydrogenase (ubiquinone) activity	6.19E-21	29.99	7.99E-18
GO:0032981 mitochondrial respiratory chain complex I assembly	2.67E-20	24.58	4.10E-17
GO:0005829 cytosol	4.67E-20	2.46	5.90E-17
GO:0070062 extracellular exosome	1.55E-19	2.65	1.96E-16
GO:0003723 RNA binding	2.70E-15	5.11	3.44E-12

### qPCR analysis

**Fig. 4:**
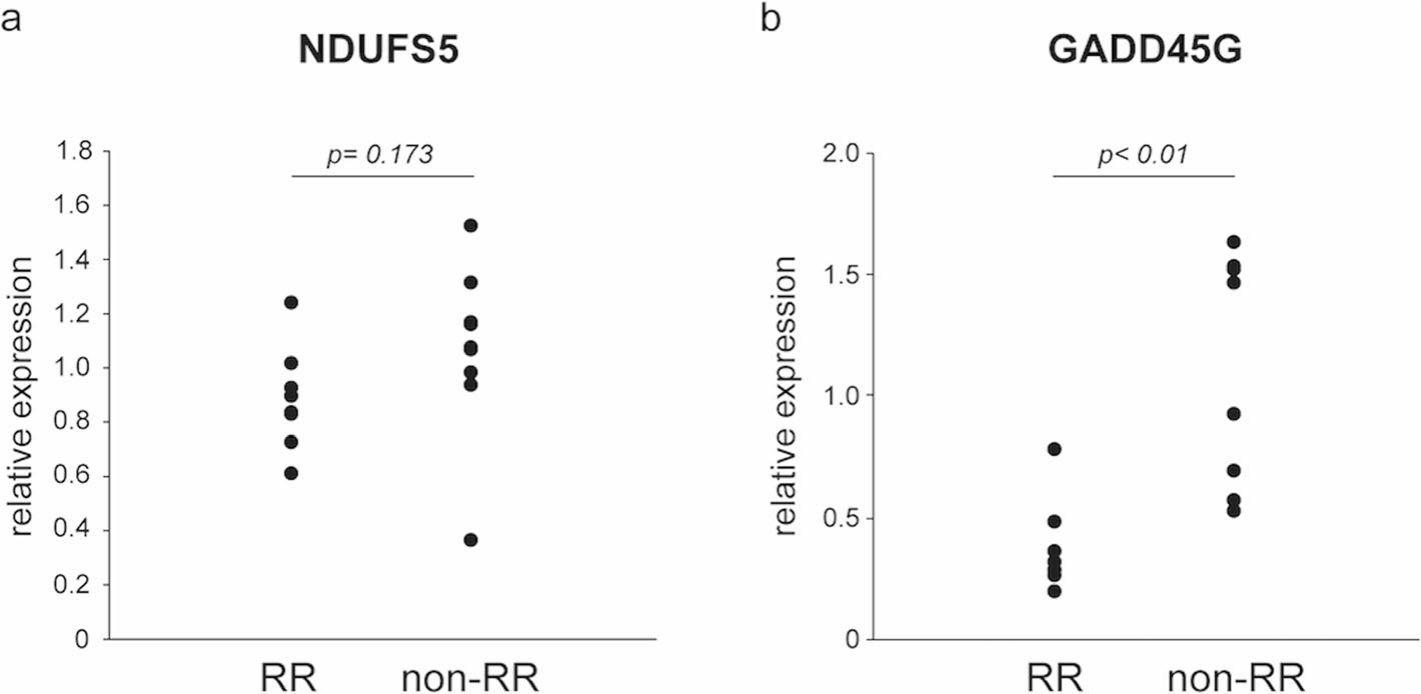
Relative fold expression levels of NDUFS5 and GADD45G, as determined by quantitative real-time PCR. Gene expression was compared between the RR and non-RR groups; results were consistent with those obtained by RNA-seq. **a**) NDUFS5 (*n* = 8 and 9 in the RR and non-RR groups, respectively), **b**) GADD45G (*n* = 8 each)

qPCR was performed with residual RNA samples. The RR group showed lower expression of *NDUFS5* and *GADD45G* than the non-RR group, consistent with the results obtained by RNA-seq (Fig. 4).

### Logistic regression analysis

Logistic regression analysis was performed to examine the influence of clinical variables including selected DEGs on LVRR (Table 4). In univariate analysis, inotrope use, *NDUFS5*, and *GADD45G* showed significant association between LVRR (inotrope use: OR 0.04, 95% CI 0.003–0.57, *P* < 0.05; *NDUFS5*: OR 0.07, 95% CI 0.01–0.92, *P* < 0.05; *GADD45G*: OR 0.28, 95% CI 0.09–0.89, *P* < 0.05). In multivariate analysis, neither of these variables showed significant correlation with LVRR, with *GADD45G* demonstrating borderline significance (OR 0.38, 95% CI 0.13–1.07, *P* = 0.067).

**Table 4:** Odds ratios (95%CI) for the association with left ventricular reverse remodeling

Variables	Univariate	Multivariate^a^	
		model 1	model 2
Age	0.99 (0.91–1.08)		
Male Sex	0.38 (0.27–5.17)		
Disease duration (months)	0.98 (0.96–1.01)		
Height (cm)	0.94 (0.81–1.09)		
Weight (kg)	0.97 (0.88–1.07)		
HR (bpm)	1.01 (0.97–1.06)		
Beta-blocker	0.88 (0.05–16.74)		
ACEI or ARB	0.38 (0.03–5.17)		
Inotropes	0.04 (0.003–0.57)*	0.002 (0.001–18.0)	0.06 (0.001–2.95)
Hb (g/dL)	1.44 (0.86–2.41)		
Cre (mg/dl)	0.05 (0.003–1.06)		
BNP (pg/ml)	1.00 (0.99–1.01)		
LVDD (mm)	0.94 (0.85–1.03)		
LVEF (%)	1.06 (0.85–1.32)		
LAD (mm)	0.96 (0.86–1.06)		
AR (moderate to severe)	1.77 (0.74–4.23)		
MR (moderate to severe)	0.61 (0.20–1.93)		
TR (moderate to severe)	0.70 (0.26–1.89)		
PAP, mean (mmHg)	0.97 (0.88–1.08)		
PCWP, mean (mmHg)	0.96 (0.84–1.10)		
RAP, mean (mmHg)	0.80 (0.57–1.12)		
CI (L/min/m^2^)	4.75 (0.35–63.67)		
PVR (Wood)	0.98 (0.53–1.83)		
*NDUFS5* (per 100FPKM)	0.07 (0.01–0.92)*	0.007 (0.001–7.24)	
*GADD45G* (per 10FPKM)	0.28 (0.09–0.89)*		0.38 (0.13–1.07)

Abbreviations: *HR* heart rate; *ACEI* angiotensin converting enzyme inhibitor; *ARB* angiotensin II receptor blocker; *Hb* hemoglobin; *Cre* creatinine; *LVDD* left ventricular diastolic diameter; *LVEF* left ventricular ejection fraction; *LAD* left atrial diameter; *AR* aortic regurgitation; *MR* mitral regurgitation; *TR* tricuspid regurgitation; *PAP* pulmonary artery pressure; *PCWP* pulmonary capillary wedge pressure; *RAP* right atrial pressure; *CI* cardiac index; *PVR* pulmonary vascular resistance

^a^Confounders with *p* values < 0.05 in each analysis group were included into the model

*p < 0.05

## Discussion

In the present study, we performed RNA-seq of myocardium tissue samples from patients with advanced NIDCM and compared the gene expression profiles of those with and without LVRR. Clinically, patients with LVRR showed markedly better renal function. They also needed significantly less inotrope support, surgical biopsy and VAD implantation through shorter disease duration than those without LVRR. The major findings were that (i) several genes including *LOC100506295*, *NDUFS5* along with those encoding other NDUF subunits, *GADD45G*, and some genes encoding ribosomal protein subunits were upregulated in the non-RR as compared to the RR group; and (ii) genes associated with the mitochondrial respiratory chain and ribosome were enriched in the non-RR relative to the RR group.

Other than *LOC100506295* whose function is unknown, *NDUFS5* was most highly correlated with LVRR. *NDUFS5* encodes a subunit of mitochondrial respiratory chain complex I [28]. Previous studies have shown that disturbance of mitochondrial function can cause heart failure [29–31], which is thought to result from increased reactive oxygen species (ROS) production and apoptosis [32]. Decreased complex I activity is also associated with increased ROS production [33–35], which can contribute to the progression of heart failure. Another report demonstrated that AF-HF001, a clinical drug candidate for heart failure, reversed the up-regulation of NDUFS5 expression in H9c2 rat cardiomyocytes and attenuated ROS production and myocardial cell apoptosis [36]. These observations suggest that *NDUFS5* along with other NDUF subunits of mitochondria complex I play a crucial role in the pathophysiology of heart failure. This is supported by the difference in *NDUFS5* expression between the RR and non-RR groups as well as the enrichment of genes related to mitochondrial respiratory chain observed in the present study. Thus, *NDUFS5* and mitochondrial complex I subunits may serve as predictive biomarkers for LVRR.

Inborn mitochondrial abnormality, i.e. mitochondrial disease (MD), can involve myocardium and induce cardiac dysfunction through disrupted mitochondrial respiratory chain functions [37]. In addition, even isolated cardiac phenotype is found in MD [38]. Thus, inherent but underdiagnosed MD might be included in the study population. This notion is supported by the previous report that 31% of MD patients represented cardiac conditions including cardiac dysfunction before the diagnosis [39]. In the present study, we did not examine mutations of mitochondrial DNA nor mitochondria-related nuclear DNA. However, given the lack of typical clinical signs in the participants of our study, MD with manifest cardiomyopathy such as Mitochondrial encephalomyopathy, lactic acidosis, and stroke-like episodes (MELAS), Chronic progressive external ophthalmoplegia (CPEO), and Myoclonic epilepsy with ragged-red fibers (MERRF) are unlikely. Further study is warranted to determine the prevalence of MD in this population.

Apoptosis is another hallmark of mitochondrial function, with mitochondrial permeability transition pore opening to trigger intrinsic apoptosis pathway. Thus, disruption of mitochondrial function can enhance cardiomyocyte apoptosis, leading to progression of heart failure [40, 41]. In this study, *GADD45G* was found to be strongly correlated with LVRR. *GADD45G* is a member of the GADD45 family of proteins that are involved in p38 mitogen-activated protein kinase-dependent cell death [42]. Recent studies have reported that *GADD45G* overexpression in mice induced cardiomyocyte apoptosis, fibrosis, LV dysfunction, and heart failure, whereas *GADD45G* deletion conferred resistance to ischemic injury and cardiomyocyte apoptosis [43]. In our study, *GADD45G* was more highly expressed in non-RR as compared to RR patients, suggesting that *GADD45G* upregulation is linked to induction of apoptosis and consequently, a reduced probability of LVRR.

Our study had two novel findings as compared to previous transcriptomic analyses of LVRR. First, we identified markers for predicting LVRR by comprehensive transcriptome analysis of single samples obtained prior to treatment. Some studies showed associations between certain mRNAs or miRNAs and myocardial recovery or subsequent VAD removal after LVAD implantation [13, 14, 44]. However, these studies analyzed only limited number of cardiac gene expression. Dhar et al. performed RNA-seq for the cardiac samples obtained from the patients with non-ischemic advanced heart failure requiring LVAD implantation, and showed that the expressions of myosin light chain kinase and interleukin-6 genes were significantly higher in the LVAD responders as compared to the non-responders. In this study, however, only 95 genes associated with heart failure were examined [44]. RNA-seq in the present study performed a comprehensive transcriptome analysis, thereby demonstrating previously unknown, novel mechanisms of LVRR.

Secondly, cardiac biopsy can reveal not only histological abnormalities but also the potential reversibility of cardiac function, which increases its clinical utility for NIDCM. Cardiac biopsy—particularly by the transcatheter method—is associated with risks such as perforation or idiopathic valvular regurgitation, which has thus far limited the diagnostic yield. However, taking into consideration information on the reversibility of cardiac function in NIDCM can facilitate clinical decision-making based on biopsy samples. Favorable expressions of the specific genes possibly make us advance aggressive medical treatment to avoid VAD implantation, or even to introduce VAD as a bridge to recovery strategy. By contrast, unfavorable expressions would lead us to prompt introduction of VAD as a bridge to transplant strategy.

The present study had several limitations. First, this study was carried out at a single institution with a small number of samples. Previous studies examining myocardial samples from severely failing hearts could collect only around 10 samples for each treatment group [14, 20, 44]. These facts probably reflect the small population of patients whose heart failure status are severe enough to assess pathological specimen of the heart. Multicenter study will overcome both the possible bias from the single-centered study and the less-powered and non-validated results due to small sample size. Second, the issue of small sample size also influenced the results of logistic regression analyses. The model was not established with more than three independent variables due to small number of samples. Even with the challenge, *GADD45G* demonstrated almost significant predictive value for LVRR in the multivariate model, which highlights the strong contribution of the gene. Reconfirmation of the results with more large samples is needed to show clinically definite significance of *NDUFS5* and *GADD45G*. Third, there were unequal number of females and males. Some transcripts might be expressed in a gender specific manner and serve as a bias for the results of the present study. Large-scale and preferably randomized control study is required to resolve this issue. Fourth, adopted treatment strategy differed between the groups. There were more LVAD implantation in the non-RR group, however, this difference was not arbitrary and resulted from the continuing optimal treatment for severe heart failure. Mechanical ventricular support is the most potent cardiac unloading therapy and is thus likely to promote LVRR more efficiently. Nonetheless most patients with LVRR implantation fell upon the non-RR group in the present study, underscoring that the non-RR group actually had little chance to recover cardiac function even after maximal heart failure treatment. Of note, gene expression analyses were performed on the samples prior to LVAD implantation and were not influenced by the difference in the treatment strategy. Fifth, we identified LVRR only by echocardiography in both VAD and non-VAD patients, in contrast to previous studies that defined LV recovery as removal of VAD in VAD-patients [14]. In the present study, there were two VAD patients in the RR group (showing elevations in LVEF from 18 to 39% and from 13 to 37%) who nonetheless required VAD support. However, since these patients showed at least partial recovery of cardiac function, pump flow was successfully weaned without right heart failure, aortic valve insufficiency, or readmission. Sixth, the patients had variable etiology, which included drug-induced cardiomyopathy or valvular heart disease. In fact, the etiology may not be known in some patients diagnosed as idiopathic DCM; additional studies are needed to address this issue. However, we presumed that the observed changes in gene expression are common to heart failure patients irrespective of etiology and can therefore serve as universal biomarkers. Finally, the sampled sites differed among patients—i.e., the apical and posterolateral walls were sampled by surgical and transcatheter biopsy, respectively—yet all patients showed a general decrease in cardiac function, implying homogeneous pathophysiology throughout the heart.

## Conclusions

In conclusion, we identified novel genes that are correlated with LVRR by RNA-seq in myocardium tissue samples from patients with advanced NIDCM. *NDUFS5* and *GADD45G* are potential biomarkers for predicting whether patients will develop LVRR, which can inform clinical decision making regarding VAD implantation and/or removal. These findings add value to conventional cardiac biopsy and can potentially improve the treatment of heart failure patients.

## Data Availability

The datasets generated and analyzed during the current study are available in the BioProject of the DNA Data Bank of Japan (DDBJ) repository (https://www.ddbj.nig.ac.jp/bioproject/index-e.html), accession ID PRJDB5368.

## Abbreviations

CPEO: Chronic progressive external ophthalmoplegia
CRT: Cardiac resynchronization therapy
DEG: Differentially expressed gene
FPKM: Fragments per kilobase of exon per million fragments
*GADD45g*: *Growth arrest and DNA-damage-inducible protein 45G*
LV: Left ventricle
LVAD: Left ventricular ventricular assist device
LVEF: Ejection fraction
LVRR: Left ventricular reverse remodeling
MELAS: Mitochondrial encephalomyopathy, lactic acidosis, and stroke-like episodes
MERRF: Myoclonic epilepsy with ragged-red fibers
miRNA: microRNA
*NDUFS5*: *Nicotinamide adenine dinucleotide ubiquinone oxidoreductase subunit S5*
NGS: Next generation sequencing
NIDCM: Non-ischemic dilated cardiomyopathy
PCA: Principal component analysis
qPCR: Quantitative real-time polymerase chain reaction
RNA-seq: RNA sequencing
ROC: Receiver operating characteristic
ROS: Reactive oxygen species
RR: Reverse remodeling
VAD: Ventricular assist device
